# The Effects of Non-Thermal Atmospheric Pressure Plasma treated Titanium Surface on Behaviors of Oral Soft Tissue Cells

**DOI:** 10.1038/s41598-018-34402-x

**Published:** 2018-10-29

**Authors:** Won-Seok Jeong, Jae-Sung Kwon, Eun-Ha Choi, Kwang-Mahn Kim

**Affiliations:** 1 Department and Research Institute of Dental Biomaterials and Bioengineering, Yonsei University College of Dentistry, 50-1 Yonsei-ro, Seodaemungu, Seoul, 03722 Korea; 2 BK21 Plus Project, Yonsei University College of Dentistry, 50-1 Yonsei-ro, Seodaemungu, Seoul, 03722 Korea; 3 Plasma Bioscience Research Center, Kwangwoon University, Seoul, 01897 Korea

## Abstract

Here, we investigated the possible use of the technology known as non-thermal atmospheric pressure plasma on integration and control of cytokine release of soft tissue on titanium surface. After NTAPP was applied to titanium samples, changes of surface characteristics were measured as topographical features, contact angle, surface tension, and with X-ray photoelectron spectroscopy (XPS). Protein absorption was evaluated using a bovine serum albumin absorption assay. The attachment, viability, morphology, proliferation, and cytokine release of soft tissue on titanium were assessed. No change in topographical features was observed between control and NTAPP-treated groups. However, NTAPP treatment resulted in significant lowering of the contact angle for polar and non-polar liquids and increase of surface tension. Protein absorption was significantly enhanced on the NTAPP-treated samples. Normal soft tissue attachment was improved on the NTAPP-treated groups with good viability. Cellular morphology was improved in NTAPP-treated groups whereas cellular proliferation was not enhanced. There was a significant reduction in the amounts of cytokine release for inflamed IHOK and hTERT-hNOF on the NTAPP-treated groups; except for IL-8 for IHOKs. This study demonstrates that surface functional consequences by NTAPP exposure enhanced behavior of oral soft tissue cells without topographical change.

## Introduction

The restoration of missing teeth by using dental implants is a common strategy^1^. However, although high success rates of dental implants have been extensively reported, approximately 5–10% failure rate is also observed^2–5^. This failure may occur because of the inability of soft tissue integration to induce well-established osseointegration or from the disruption of established osseointegration that^2–4^ may occur owing to peri-implantitis^6^.

The success of dental implants is reliant upon the integration of soft tissue surrounding the titanium abutment. Following dental implant surgery, compact integration between the titanium abutment and the soft tissue acts as protective barrier against oral bacterial infection^7^ and may also affect osseointegration^8^. Notably, many studies have reported that surface chemical^9,10^ or topographical change^11,12^ may enhance soft tissue integration through means such as facilitating fibroblast and epithelial cell attachment on the material surface.

Peri-implantitis is common disease that occurs surrounding the dental implant and represents an inflammatory process that affects the failure of dental implants and loss of supporting bone^13^ However, this reaction is necessary to an effective immune response. Upon bacterial invasion, tissues in the body secrete cytokines that attract leucocytes and neutrophils and induce the inflammatory process^14^. Cytokines play important roles in the pathogenesis as well as in tissue homeostasis of many infectious diseases. For example, the inflammatory reaction of periodontal tissue may be induced by a variety of cytokines^15^; in turn cytokine upregulation during the inflammatory process may play a role in wound healing as well^16,17^. In particular, inflammatory cytokines such as IL-1ß, IL-6, and IL-8 are present in inflamed periodontal tissues. However, their overproduction can lead to tissue destruction^18^. Therefore, the control of cytokine release of inflamed soft tissue is important. Currently, a gold standard for treatment of peri-implantitis does not exist. It seems that so far mechanical treatments cannot achieve complete decontamination of the implant surfaces. Because, even if bacteria is decontaminated using physical method, implant surface was more roughend and therefore give to chance of more bacterial attachment^19^. Therefore, effective method for treatment for peri-implantitis with no affect to surface roughness was desired.

In the biomedical field, non-thermal atmospheric pressure plasma (NTAPP) has been studied to determine its effects on various cells and biomaterials^20–24^. NTAPP has been extensively utilized on various titanium implants to facilitate osseointegration, inhibition of bacterial attachment, and tooth bleaching without topographical change^25^. Additionally, NTAPP treatment directly on cells has been shown to result in enhanced soft tissue cell activity^10^. However, although the importance of controlling cytokine release in inflamed soft tissue is well understood, the biological effects of NTAPP exposure on inflamed gingival soft tissue are poorly studied. Most studies have focused on attempts to enhance the viability, attachment, and proliferation of normal cells^20,21^. It also remains uncertain whether NTAPP-treated titanium might simultaneously enhance soft tissue attachment, which is associated with the clinical success of implants, while also positively impacting the inflamed soft tissues that are present in the majority of patients with symptoms necessitating implants.

To address these issues, we investigated the biological activities of normal and inflamed soft tissue cultured on control and NTAPP-treated titanium in this study. The null hypothesis was; 1) there would be no difference in integration of normal soft tissue between control and NTAPP-treated titanium, 2) there would be no difference in cytokine release of inflamed soft tissue between control and NTAPP-treated titanium.

## Material and Methods

### Specimen preparation

This study used commercially pure titanium (cp-Ti, Grade IV) discs with 13 mm diameter and 1 mm thickness. The disks were provided by Osstem Implant (Pusan, Korea). Titanium discs were polished on a polisher equipped with #400, #800, #1,200, and #2,000 grit SiC paper (Polisher and Grinder, ECOMET III, Buehler, USA). All Ti specimens were cleaned ultrasonically in acetone, ethanol, and distilled water for 15 min each. Samples were the dried at room temperature and sterilized using an autoclave.

### Nonthermal atmospheric pressure plasma (NTAPP)

**Figure 1:**
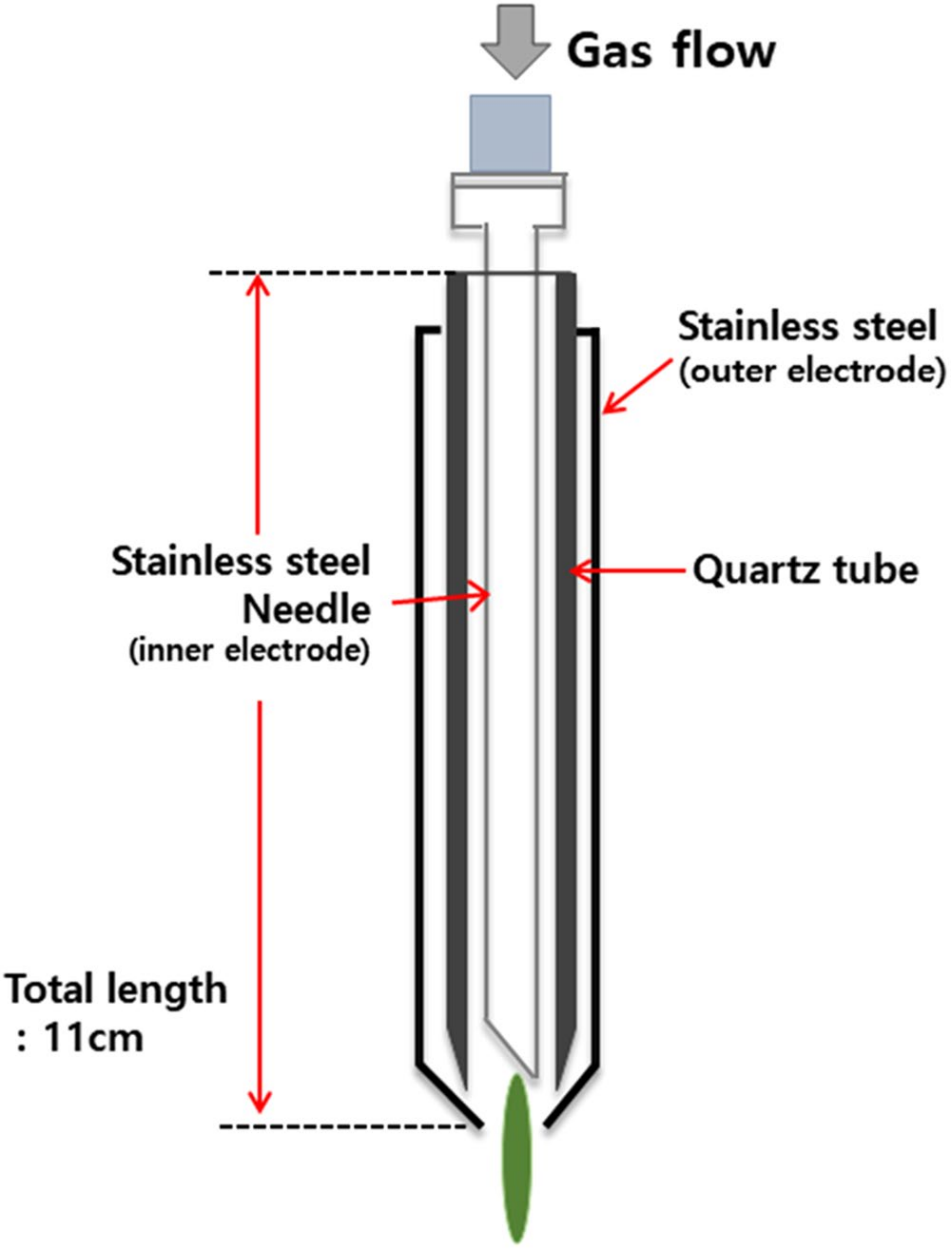
Schematic diagram of the non-thermal atmospheric pressure plasma device (provided by the Plasma Bioscience Research Center, Kwangwoon University, Seoul, Korea).

NTAPP, developed by Plasma Bioscience Research Center (Kwangwoon University, Seoul, Korea), was used in this experiment (Fig. 1). Titanium samples were exposed to NTAPP using the method and duration that was determined by previous studies^26^. The plasma was formed by passing 5 L/min of air through an NTAPP device. The maximum discharge voltage and discharge current for the system were 2.24 kV and 1.08 mA, respectively, yielding around 2.4 W of power. It consisted of a stainless steel inner electrode that was 1.2 mm in depth and 0.2 mm in thickness, along with a 3.2 mm-deep quartz dielectric component. The mirror polished titanium specimens was treated by plasma with 3 mm distance between the device tip and specimen for 10 min.

### Surface characteristics

The surface roughness of the control and NTAPP-treated groups was measured using an optical profilometer (Contour GT, Bruker, Tuscon, AZ, USA). The average of surface roughness (Ra and Sa values, μm) was confirmed using Vision64 software (Bruker, Tuscon, AZ, USA). Surface chemical composition of the control and NTAPP-treated groups was confirmed using X-ray photoelectron spectra (XPS; K-alpha, Thermo VG Scientific, Waltham, MA, USA). A monochromatic Al Kα source was operated as the X-ray source (Al Kα line: 1486.6 Ev). The binding energy was referenced and calibrated to the C1s peak at 284.8 Ev. Detailed scans were taken for the C1s, O1s, N1s, and Ti2p regions. The contact angle and surface tension of the control and experimental groups were assessed using electro optics (Phoenix-300, SEO, Seoul, Korea). Specifically, 10 μL distilled polar (water) and non-polar (ethylene glycol, Sigma-Aldrich. St. Louis, MO, USA) liquid was dropped on the center of titanium specimens and left at room temperature. After 10 s, an image of the contact angle was measured and surface energy was calculated using Image XP (ver.5.9, SEO, Suwon, Korea) according to the Owens-Wendt method.

### Protein absorption assay

Bovine serum albumin (BSA) was used as a model protein. A total volume of 200 μL protein solution (100 µM; 1 mg/mL in phosphate-buffered saline [PBS], pH 7.4) was dropped and spread on titanium surfaces. After 4 h incubation under humidified conditions at 37 °C and 5% CO_2_, protein attachment was assessed by adding 200 μL bicinchoninic acid from a Micro BCA total protein assay kit (Pierce Biotechnology, Inc., Rockford, IL, USA). Samples were further incubated at 37 °C for 2 h and optical density was confirmed at 562 nm using Epoch microplate reader (Biotek Instruments, Winooski, VT, USA). The rate of protein absorption was calculated as the percentage of albumin absorbed to the sample surface relative to the total amount using a BSA standard curve provided with the kit.

### Cells and cell culture

Immortalized human oral keratinocytes (IHOK) and immortalized human gingival fibroblasts (hTERT-hNOF) were used in this study. Both cell types were provided by the Department of Oral Pathology, Oral Cancer Research Institute, Yonsei University College of Dentistry, Seoul. Briefly, HPV-Immortalized HOKs were derived by transfecting normal human gingival epithelial cells with the PLXSN vector containing the E6/E7 open reading frames of HPV type 16, following methods previously described^27^. hTERT-hNOF cells were derived from gingival fibroblasts that were primary-cultured from healthy human adults and transfected with the puromycin-resistant retroviral vector plpc-hTERT (Clonetech Laboratories, Palo Alto, CA, USA)^28^. Previous studies have confirmed that sub-culturing beyond the 90th passage could be performed without signs of replicative senescence and the feasibility of biocompatibility evaluation has been demonstrated^28,29^. Both cell types were prepared from 90% confluent cells for the experiments in this study. All culture media used in this study comprised Dulbecco’s modified Eagle medium/F-12 nutrient mixture Ham’s 3:1 mixture (Welgene, Daegu, Korea) with 10% fetal bovine serum (Gibco, Grand Island, NY, USA) and 1% antibiotics (penicillin/streptomycin, Gibco). Lipopolysaccharide (LPS) was most frequently used to mimic periodontal disease; upon soft tissue cell treatment with bacterial LPS, the soft tissues then release inflammatory cytokines^30^. In this study, IHOK and hTERT-hNOF cells inflamed using 1 μg/mL LPS and normal cells not treated with LPS were used.

### Cell attachment

The numbers of normal and inflamed soft tissue cells attached on the surface of control and NTAPP-treated groups were confirmed using a water soluble tetrazolium (WST) assay (Daeil Lab Services Co., Ltd., Seoul, Korea). The method used was similar to that of other WST or MTT-based methods based on the change into yellow/orange color owing to the reduction of the reagent by viable cells. Both cell (3 × 10^4^/100 μL) were individually placed on each titanium specimen in the each standard 12-well culture plates (SPL, Daegu, Korea). Both cells were cultured under humidified conditions at 37 °C and 5% CO_2_. After 4 h of incubation, supernatants from each cell type were collected for the cytokine release assay. Unattached cells were washed away using phosphate buffered saline (Gibco) and WST assay solution was added to each group. Following incubation for a further 2 h at 37 °C, optical density was then measured at 450 nm with reference optical absorbance taken at 650 nm using the Epoch microplate reader. Cell attachment results are shown as relative percentage to the control.

### Cell viability

Cell viability of the normal and inflamed soft tissue cells cultured on control and experimental groups was confirmed by staining the cells using calcein and ethidium homodimer-1 (LIVE/DEAD^TM^ Viability/Cytotoxicity Kit, Invitrogen, Grand Island, NY, USA). Briefly, 3 × 10^4^/100 μL of each cell type was placed on top of the titanium specimens in standard 12-well culture plates. Both cell types were cultured under humidified conditions at 37 °C and 5% CO_2_. After 4 h, cell culture media was then removed from each well and cells were washed with phosphate buffered saline (PBS; Gibco, Grand Island, NY, USA). Combined LIVE/DEAD assay reagents, using optimized concentrations, were added directly to the cells on the titanium. The cells were then observed using a confocal laser microscope (LSM700, Carl Zeiss, Thornwood, NY, USA), with live cells visible as green and dead cells as red color.

### Cell morphology

The morphology of attached normal and inflamed soft tissue cells on the control and experimental groups was measured using a fluorescent dye and a confocal laser microscope. After 4 h culture as described for the cell viability assay, both cell types were stained with DAPI (providing blue coloration for nuclei; Invitrogen) and rhodamine-phalloidin (red coloration for actin filaments; Invitrogen). The stained cells on titanium were then assessed using the LSM700 confocal laser microscope. Quantitative assessment of cell morphology was performed using Image J software (NIH, Bethesda, MD, USA) with parameters of cell area and Feret’s diameter.

### Cell proliferation

The proliferation of normal and inflamed soft tissue cells on the control and experimental groups was investigated using BrdU incorporation during DNA synthesis. Briefly, 3 × 10^4^/100 μL each cell type was seeded on the top of titanium specimens in standard 12-well culture plates. Both cell types were cultured under humidified conditions at 37 °C and 5% CO_2._ Following 4 h of culture, 100 μL BrdU solution (100 mM) (Roche Applied Sciences, Penzberg, Germany) was added on each group and further incubated for a proliferation period of 4 h. Both cell types with incorporated BrdU were reacted with anti-BrdU conjugated with peroxidase for 90 min. Finally, tetramethylbenzidine was added for color development. The optical density was quantified using an Epoch microplate reader at 370 nm. The proliferation results are shown as relative percentage to the control.

### Cytokine assay

Both cell types were treated with LPS (026:B6, Sigma-Aldrich). IHOK and hTERT-hNOF (3 × 10^4^/100 μL cells) were treated using 1 μg/mL *Escherichia coli* and incubated at 37 °C and 5% CO_2_ for 24 h^30^. Cell supernatants were harvested as described in section 2.6 and analyzed using the cytokine release test. Briefly, interleukin-1β (IL-1β), interleukin-6 (IL-6) or interleukin-8 (IL-8) pre-coated 96-well plate (Koma Biotech Inc., Seoul, Korea), covered with the provided plate sealer, and incubated at room temperature for 2 h. After washing with PBS, plates were incubated at room temperature for 2 h with 100 μL detection antibody, washed, incubated for 30 min with the color development antibody, washed, and incubated with streptavidin-HRP conjugate for 10 min, with 100 μL stop solution then added to halt color development. The absorbance of each well was measured at 450 nm using an Epoch microplate spectrophotometer and levels of IL-1β, IL-6, and IL-8 were determined using a calibration curve drawn using dilutions of standard recombinant human IL-1β, IL-6, or IL-8 (Koma).

### Statistical analysis

The statistical analysis of the results of surface roughness, contact angle, surface tension, and protein absorption was carried out using a paired Student’s *t*-test. Cell attachment, proliferation, morphology (Image J) and cytokine release tests were analyzed using one-way analysis of variance (ANOVA) combined with post-hoc test using Tukey’s method. Statistical analysis was carried out using IBM SPSS statistics 20 program (IBM, Armonk, NY, USA). Statistical significance was accepted at a confidence level of 95% (*p* < 0.05).

## Results

### Surface characteristics

**Figure 2:**
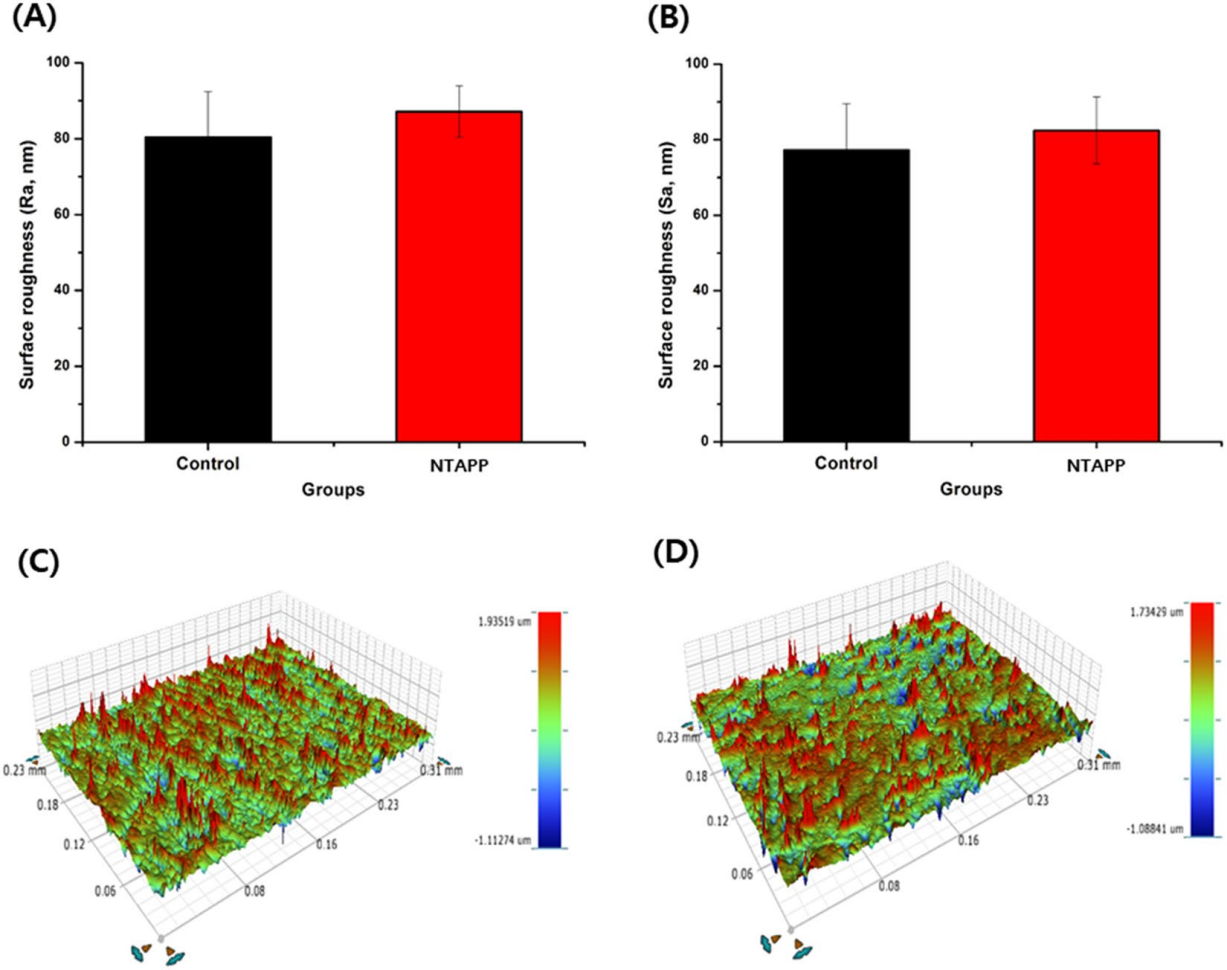
Results of surface topography of titanium surfaces. Surface roughness parameters, (**A**) Ra and (**B**) Sa were quantitatively measured and the results were compared between groups. (**C**,**D**) Three-dimensional images of (**C**) control and (**D**) experimental group titanium surfaces. The mean values and standard deviations are shown and all statistical significance was declared at *p* < 0.05.

The surface roughness of control and NTAPP-treated groups was compared using an optical profilometer (Fig. 2). There was no significant difference in surface roughness (Sa, Ra) values between the control and NTAPP-treated groups. Ra values of the control and NTAPP-treated groups were 80.41 ± 12.00 nm and 87.14 ± 6.74 nm, respectively (Fig. 2A); Sa values were 77.29 ± 12.29 nm and 82.49 ± 8.87 nm (Fig. 2B). In addition, no significant difference in terms of the three-dimensional (3D) images of the titanium surfaces were observed prior to or following NTAPP treatment (Fig. 2C,D).

**Figure 3:**
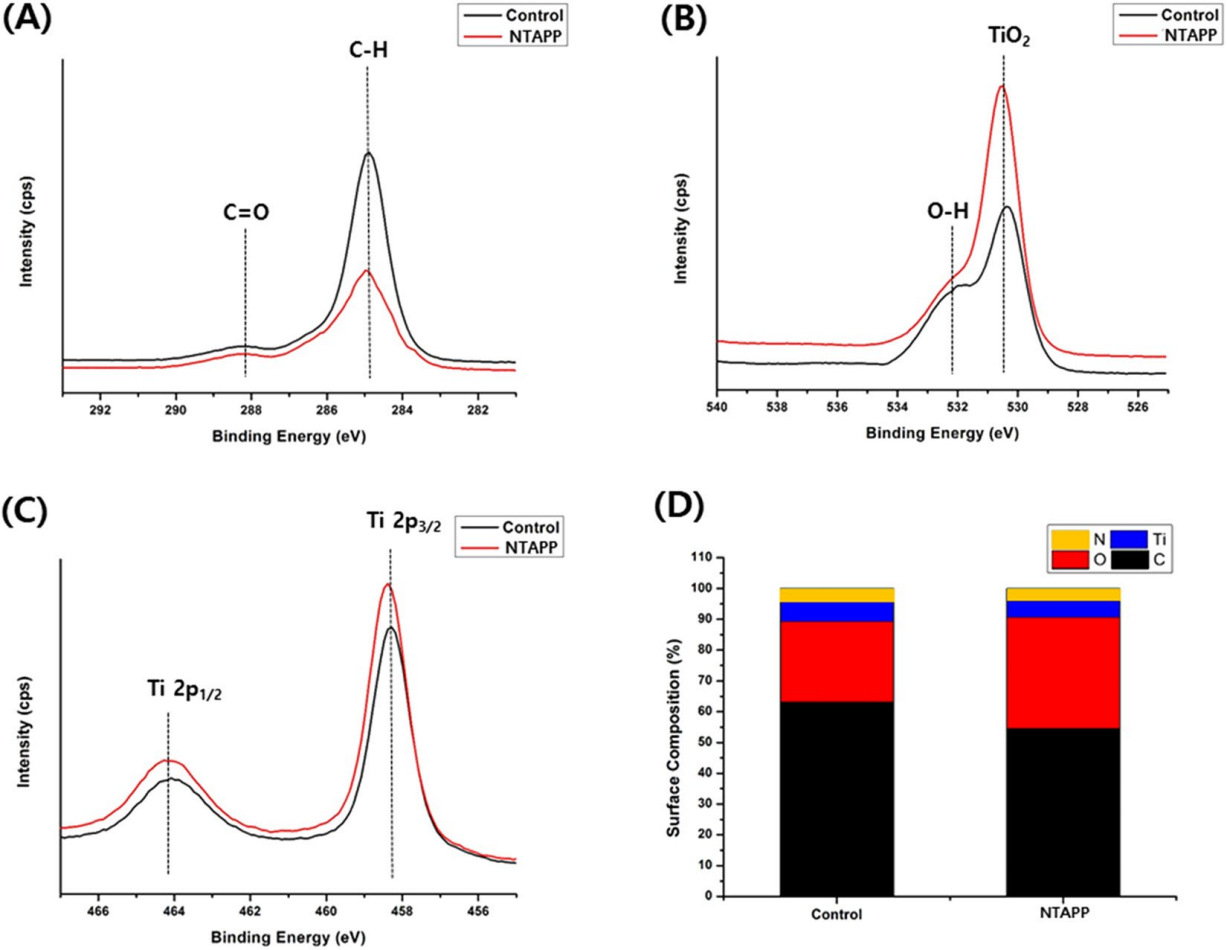
Surface chemistry of the tested samples. High resolution (**A**) C1s, (**B**) O1s, and (**C**) Ti2p spectra acquired from the test sample surfaces. (**D**) Atomic percentage of test sample surfaces.

As shown in Fig. 3, chemical shifts and changes in the chemical composition for the control and NTAPP-treated groups were confirmed by XPS. Both groups showed C1s peaks with the dominant peak corresponding to the hydrocarbon (-CH) at a binding energy of 284.9 eV (C_1_), although this decreased after NTAPP treatment (Fig. 3A). Additionally, there was a small decline in the C_2_ peak (288.1 eV) corresponding to carbon-oxygen (C-O) bonds after NTAPP treatment. Similarly, after NTAPP treatment, the carbon atomic percentages were lower than before treatment (Fig. 3D). With respect to the O1s spectra, the peak for TiO_2_ at a binding energy of 530.5 eV was evident for each group although the peak intensity increased with NTAPP treatment (Fig. 3B). Furthermore, the peak of the hydroxyl group (-OH) on each group at a binding energy of 532.1 eV also increased with NTAPP treatment. These changes implied an increase of the oxygen atomic percentage with NTAPP treatment of titanium specimens (Fig. 3D). Finally, the peaks corresponding to Ti2p 1/2 and Ti2p 3/2 components can be observed in Fig. 3C, which were evident at binding energies ranging from 458.8 to 464.9 eV.

**Figure 4:**
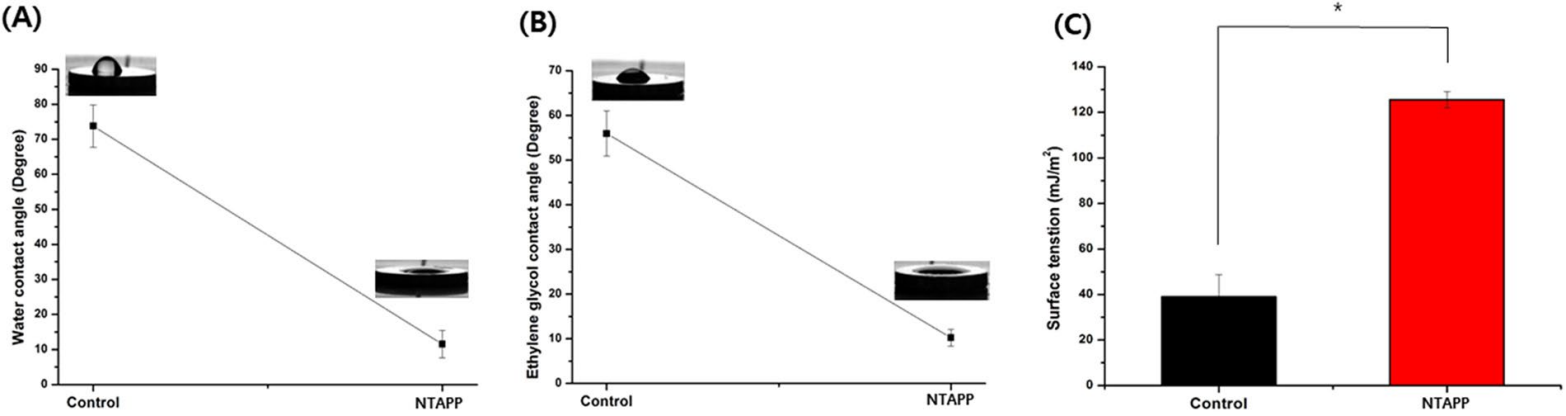
Changes in the (**A**) water and (**B**) ethylene glycol contact angles on titanium surfaces before or after plasma treatment as assessed using typical droplet images. (**C**) Surface tension was measured according to the Owens-Wendt method. The mean values and standard deviations are shown and all statistical significance was declared at *p* < 0.05.

The contact angle control and NTAPP-treated groups was measured using water and ethylene glycol contact angle analysis (Fig. 4). There was a significant difference of contact angle and surface tension between the control and NTAPP-treated groups (*p* < 0.05). The water contact angle of the control and NTAPP-treated groups was 73.77 ± 6.05° and 11.53 ± 3.96°, respectively (Fig. 3A). In particular, in the NTAPP-treated groups, hydrophilicity increased markedly with decreased contact angle (Table 1). The ethylene glycol contact angle of control and NTAPP-treated group was 55.93 ± 5.03° and 10.23 ± 1.89°, respectively (Fig. 4B). Furthermore, there was a significant change in surface tension between the control and NTAPP-treated groups (Fig. 4C, *p* < 0.05), which shifted from 39.02 ± 9.75 to 125.57 ± 3.51 mJ/m^2^, respectively (Table 1).

**Table 1:** Water and ethylene glycol contact angles of the titanium surface before and after NTAPP exposure.

Test groups	Liquid (degrees)		Surface tension (mJ/m^2^)	Dispersive force (mJ/m^2^)	Polar force (mJ/m^2^)
	Water	Ethylene glycol			
Before NTAPP exposure	73.77 ± 6.05	55.93 ± 5.03	39.022 ± 9.75	0.61 ± 0.94	35.74 ± 11.35
After NTAPP exposure	11.53 ± 3.96	10.23 ± 1.89	125.57 ± 3.51	2.49 ± 0.4	125.51 ± 3.51

### Protein absorption

**Figure 5:**
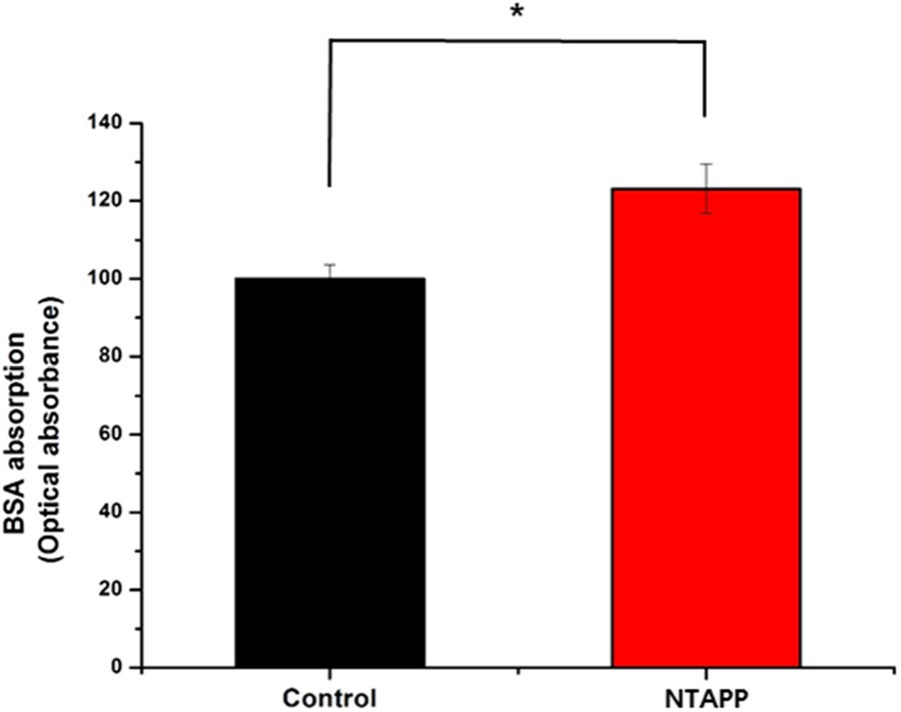
Attachment of bovine serum albumin (BSA) on the control and plasma-treated titanium samples. *p < 0.05 for comparisons between the indicated groups.

The protein absorption of the control and NTAPP-treated groups were confirmed using a BSA model (Fig. 5). Following NTAPP treatment, the amount of attached BSA on titanium specimens was significantly higher than that on the control group (*p* < 0.05). BSA adhesion on the control and NTAPP-treated groups was 100.00 ± 3.59% and 123.17 ± 6.34%, respectively

### Cell attachment and cell viability

**Figure 6:**
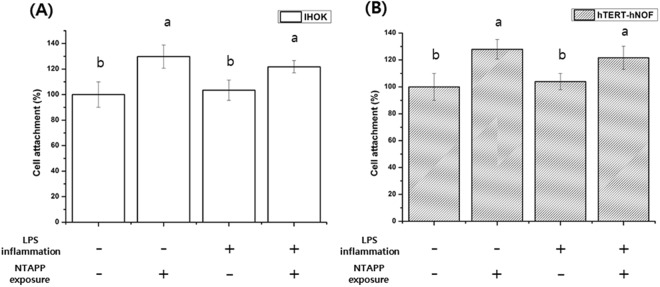
Cell attachment of (**A**) IHOKs and (**B**) hTERT-hNOFs on control and NTAPP-treated groups. The same lower case letter indicates no significant differences, whereas a different lower case letter indicates significant differences (*p* < 0.05) with greater cell attachment. Also, the mean values and standard deviations are shown.

**Figure 7:**
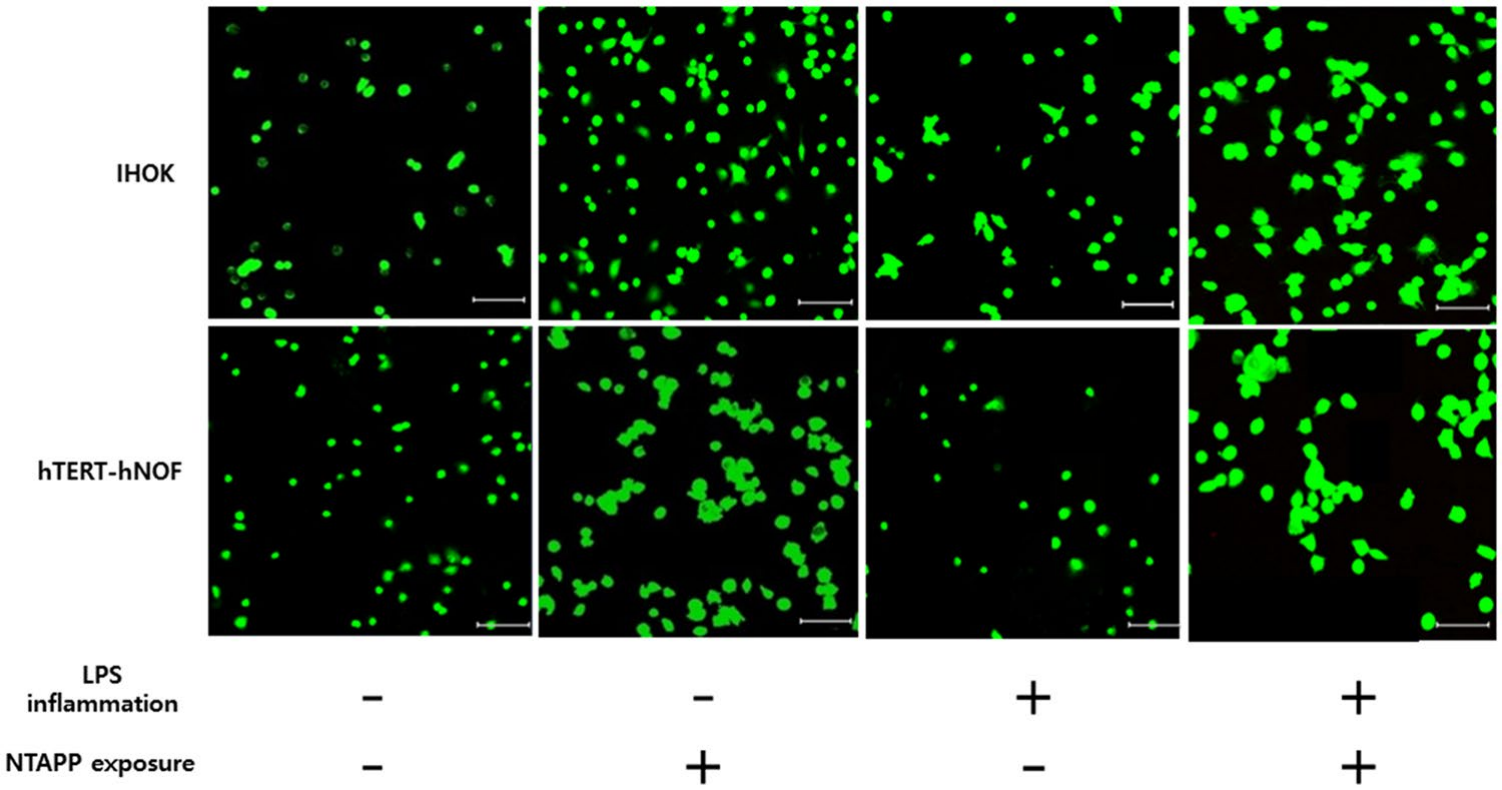
Immunofluorescence images of attached live IHOKs and hTERT-hNOFs on test samples. Scale bar = 100 μm.

The cell attachment of normal IHOKs and hTERT-hNOFs on the control and NTAPP-treated groups are shown in Fig. 6A,B. The cell attachment of normal and inflamed IHOKs on titanium showed that there was a significant increase in cell attachment with NTAPP exposure compared to the control group (*p* < 0.05). A similar change was observed in the results with hTERT-hNOFs cells (*p* < 0.05). Furthermore, improved cell viability was also observed on NTAPP-treated compared to control as confirmed using confocal microscopy. Notably, no red cells were visible on the NTAPP treated samples, indicating there was no dead cells (Fig. 7).

### Cell morphology

**Figure 8:**
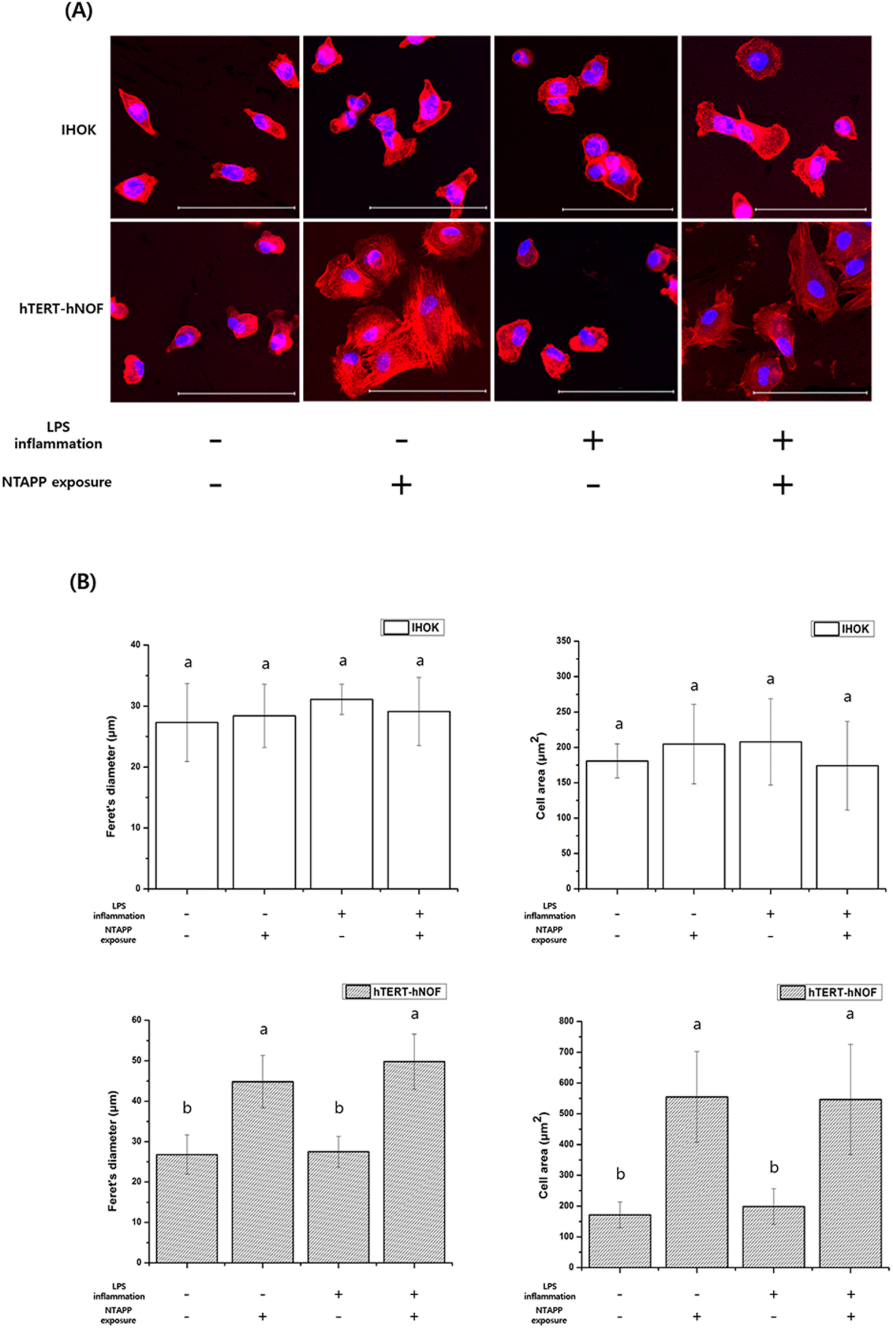
Changes in test sample morphology on test samples. (**A**) Test groups were stained with DAPI and rhodamine-phalloidin. (**B**) Comparison of cytoskeleton development using diameter and Feret’s diameter of the test samples. The same lower case letter indicates no significant differences, whereas a different lower case letter indicates significant differences (*p* < 0.05) with greater changes in cell morphology. The mean values and standard deviations are shown. Scale bar = 100 μm.

The cell morphology on titanium groups before and after NTAPP treatment is shown in Fig. 8A. The cell morphology showed a circular shape for normal and inflamed IHOKs regardless of NTAPP exposure. The value of Feret’s diameter (μm) and cell area (μm) of IHOKs also did not differ between groups (Fig. 8B). However, unlike IHOKs, the morphology of normal and inflamed hTERT-hNOFs showed a well-stretched shape on NTAPP treated titanium (Fig. 8A) whereas those on the control specimens had a relatively rounder shape with undeveloped actin filaments. The value of Feret’s diameter and cell area of hTERT-hNOFs on titanium specimens following NTAPP exposure were significantly greater than those on the control group (Fig. 8B, *p* < 0.05).

### Cell proliferation

**Figure 9:**
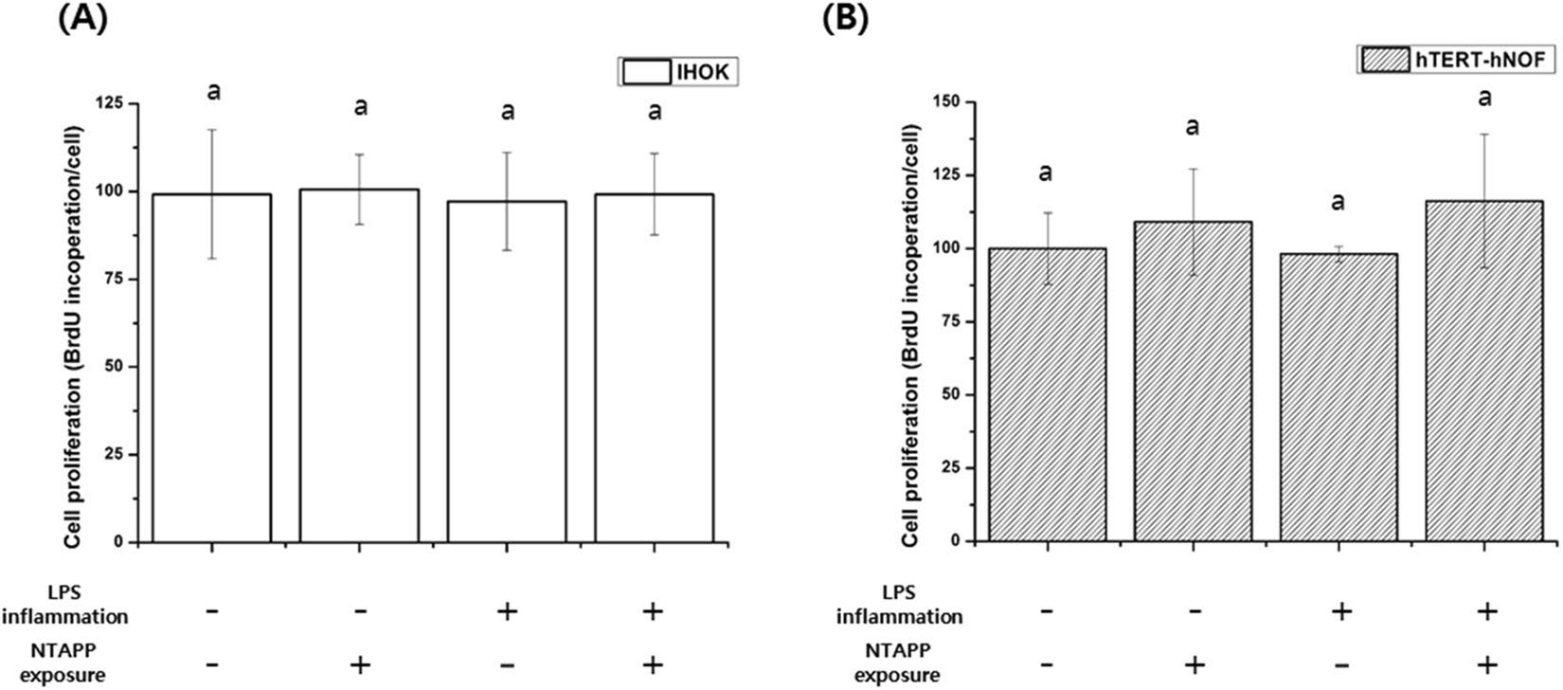
Cell proliferation rate after 24 h of test sample incubation as measured by BrdU incorporation. The same lower case letter indicates no significant differences, whereas a different lower case letter indicates significant differences (*p* < 0.05) with greater cell proliferation. The mean values and standard deviations are shown.

The cell proliferation rate of normal and inflamed cells on the control and NTAPP-treated groups was confirmed by considering the BrdU incorporation during DNA synthesis, the results of which are shown in Fig. 9. Unlike the cell attachment results, following NTAPP treatment there was no significant difference for the cell proliferation of either normal or inflamed cells for both cell types on titanium.

### Cytokine release

**Figure 10:**
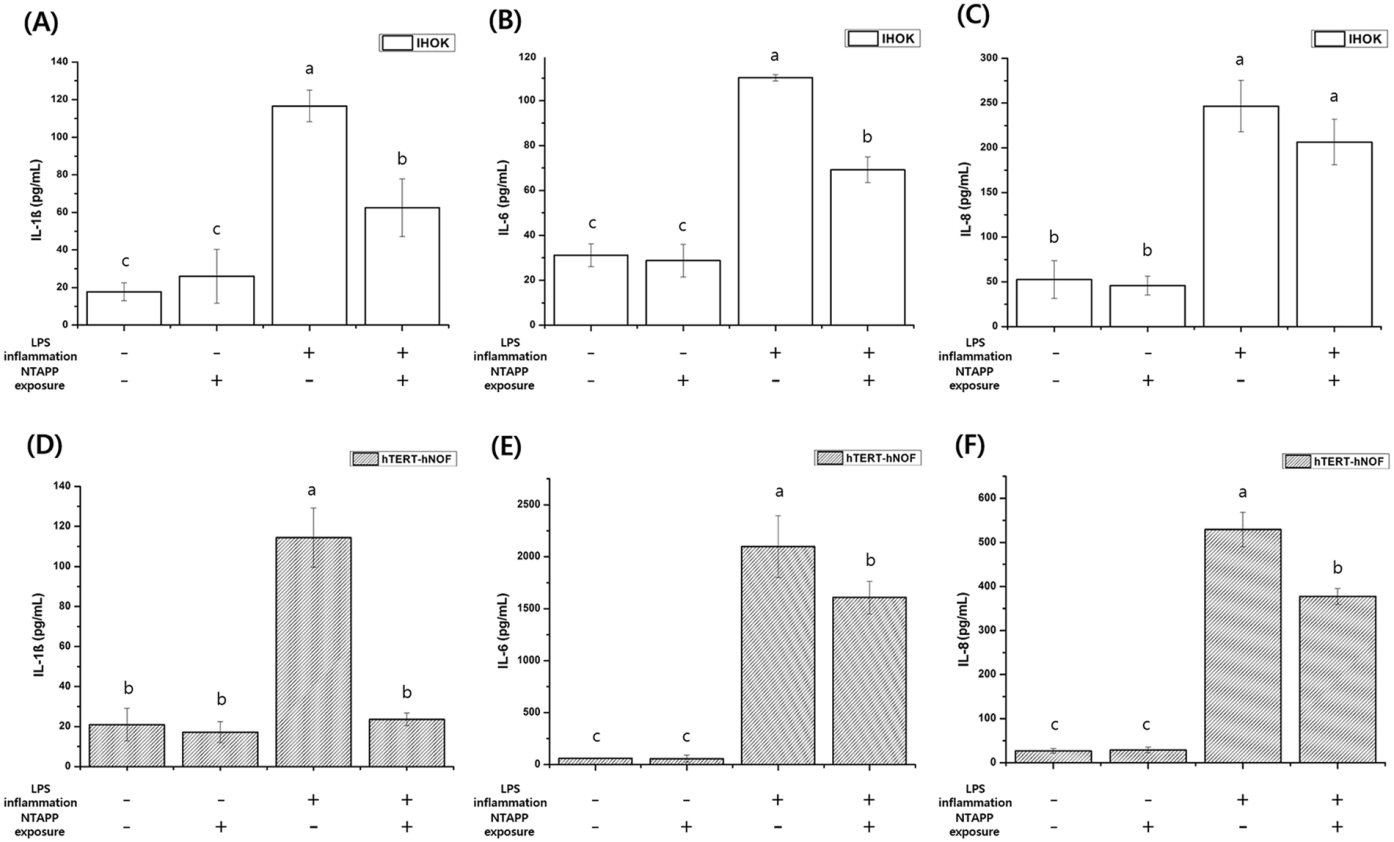
Concentration of IL-1β, IL-6, and IL-8 released from the test samples. (**A**) IL-1β, (**B**) IL-6, and (**C**) IL-8 of IHOKs; (**D**) IL-1β, (**E**) IL-6, and (**F**) IL-8 of hTERT-hNOFs. The same lower case letter indicates no significant differences, whereas a different lower case letter indicates significant differences (*p* < 0.05) with less cytokine release. The mean values and standard deviations are shown.

On the control and NTAPP-treated groups, the cytokine release of normal and inflamed cells of both cell types on titanium was investigated by measuring IL-1β, IL-6, and IL-8 expression as shown in Fig. 10. After LPS treatment, normal cells convert to an inflamed condition. Furthermore, both types of inflamed cells on titanium released high amounts of the three cytokines compare to normal cells (*p* < 0.05 for each cytokine). The cytokine release of both types of normal cells did not significantly differ based on NTAPP treatment (*p* < 0.05). Conversely, following NTAPP treatment, there was a significant difference in the cytokine release amount in inflamed cells (*p* < 0.05), with the exception of IL-8 from IHOKs.

## Discussion

Biomaterials have been commonly used in biomedical fields such as orthopaedic and dentistry fields^2–5^. In particular, integration of soft tissue on the implant surface is important factor^31,32^. Because failure or inadequate processes of soft tissue integration allow bacterial invasion onto the implant surface and therefore failure to achieve firm fixation, with subsequent implant removal or failure^33^. Theoretically, a titanium surface that has been altered through chemical treatment such as NTAPP without topographical changes may enhance the behavior of the epithelium or fibroblasts, promoting the firm integration of soft tissue^34^. Accordingly, there have been many studies related to titanium surface modification with the intent of increasing soft tissue cell attachment and concomitantly inhibiting bacterial invasion, which may result in significant bone resorption^35^. However, although several advances related to titanium surface modification have been obtained, achieving firm integration with the surrounding soft tissues over a short period time to yield increased implant success rates has remained challenging^5^.

Cytokine release as well as the integration of soft tissue affects implant success^15^ as overproduction of cytokines results in the destruction of surrounding soft tissue^18^. In addition, questions also remain related to not only the behavior of normal soft tissue but also the behavior of inflamed soft tissue on NTAPP-treated titanium surfaces.

Here, we aimed to evaluate 1) the behavior of normal soft tissue, and 2) the behavior of inflamed soft tissue cells on titanium with the use of NTAPP technology. The NTAPP device produces electrons, ions, and free radicals from the gas while remaining below body temperature and operating under normal atmosphere^20^. The use of NTAPP treatment on biomaterials has been reported by many studies^26,36^, resulting in excellent biological effect.

To demonstrate the application of NTAPP on titanium abutments, mirrored surfaces were treated under NTAPP with air gas. Surface roughness analysis using an optical profilometer confirmed that topographical features were not affected by NTAPP treatment. Surface roughness represents an important factor with respect to cellular behavior, which consequently may influence tissue formation^37^. However, in this study, we found that the topographical features were preserved with the use of NTAPP, any subsequent differences in soft tissue cell behavior on the control and NTAPP-treated groups could not be attributed to titanium surface topography but rather would derive from other factors.

We therefore performed further analysis of chemical shifts and the consequent change in chemical composition on the titanium using XPS. Although surface roughness appeared to have been preserved, with respect to chemical functional groups, NTAPP treatment of titanium decreased the amounts of hydrocarbons and increased oxygen-bonded components, such as OH and COOH^38^. It was proved that NTAPP exposure leads to the generation of reactive oxygen species (ROS), such as the hydroxyl radical^39,40^. The results demonstrated a general decrease in the carbon content and an increase in the oxygen component of the treated surface. NTAPP exposure with air gas on titanium is effective in removal of carbon composition and formation of oxygen component. These changes may underlie the observed shift of the implant surface from hydrophobic to hydrophilic following NTAPP treatment of titanium, as reflected by changes in both polar and non-polar contact angles. In addition, the surface tension on titanium was increased by use of NTAPP treatment. Contact angle and surface energy on experimental groups was increased due to reactive oxygen species (ROS) generated from NTAPP exposure. Increased ROS number from NTAPP exposure would result in a more hydrophilic condition^26^. Hydrophilicity and high surface tension after NTAPP treatment have been commonly found in the other studies^39^. Furthermore, it has been previously reported that hydrophilicity and high surface tension may be beneficial for these dental biomaterials would be more easily coated with essential cell ingredient such as blood and proteins during the implantation, thus potentially increasing the adhesion of molecules which the bonding components are not satisfied by the untreated surface^39–41^. From these results, we found that use of NTAPP did not affect topographical features and did affect removal of carbon composition. Also, these cleaned surface only shows superiority on the eukaryotic cells because prokaryotic cells attached on hydrophobic condition and eukaryotic cells attached on hydrophilic condition^42^.

In particular, it is well known that the initial absorption of blood proteins such as BSA to titanium surfaces is also an important parameter in long-term implant performance. The importance of protein absorption to biomaterial surfaces is also indicated by the total amounts of different absorbed proteins^43^ and by cell attachment. In the current study, we found that following NTAPP exposure, albumin adhesion ability to the titanium surface was significantly increased, which may have been dependent on the charge of the biomaterial surface^26^. As the percentage of hydrocarbons on a titanium surface decrease, the surface changes from electropositive to electronegative, and this phenomenon enhanced plasma protein and the extracellular matrix of the cells from adhering to the titanium implant surface and subsequent cell attachment could increase correlation with enhanced cell-protein interaction *via* Arg-Gly-Asp-binding integrin^44^. Thus, this result suggests that BSA adhesion on titanium may be highly influenced by the chemical functionality provided through the use of NTAPP treatment. However, we used one protein (BSA) to evaluate the absoprtion of the protein on titanium surface and therefore we did not account for relationship between various proteins and human plasma.

To further measure the biological efficacy of the titanium samples, in this study we utilized two types of cells; IHOKs and hTERT-hNOFs, to reflect the two-layer soft tissue composition of well-keratinized oral epithelium, which is comprised of keratinocytes as well as fiber-rich connective tissue consisting of gingival fibroblasts^34^. This chemical change effected by using NTAPP also enhanced IHOK and hTERT-hNOF attachment on the titanium sample, regardless of whether the cells were normal or inflamed. Notably, attachment of soft tissue cells on titanium comprises one of the initial events of integration and is essential for the subsequent cellular proliferation that results in protection against bacterial invasion into the bone or titanium surface^7^. Furthermore, the biocompatibility of biomaterials is essential for the acceptance of such materials and a cytotoxicity test is considered as representative of biocompatibility^45,46^. Here, we found that all of the IHOK and hTERT-hNOF cells attached on each titanium sample exhibited high viability with no cell death. The results of the cell attachment and viability tests accordingly demonstrated that the NTAPP treatment causes virtually no cytotoxicity on titanium, which is in agreement with a previous study^46^.

In addition, cell morphology is also known as an important factor for the evaluation of cell-biomaterial interactions^47^. Any changes of cell morphology such as stretching or spreading is indicative of a process of cell adhesion^48^. In our study, we identified a difference in the morphology of fibroblasts and keratinocytes. Specifically, the morphology of IHOKs showed a round shape on titanium, regardless of control and NTAPP-treated groups. As a round shape is consistent with actively growing keratinocytes^49^, this result suggests that NTAPP treatment of titanium may not have a substantial effect on cell morphology. However, the rounded cell morphology with undeveloped actin filaments observed for hTERT-hNOF cells on NTAPP-untreated groups were distinct from the stretched cells with developed actin filaments observed on NTAPP-treated titanium. This finding indicates that NTAPP treatment on titanium may enhance both the numbers and morphological features of cell attachment. The high level of actin cytoskeleton formation associated with the use of NTAPP treatment would likely result in greater cellular behavior on the biomaterial surface, which may be facilitated by the chemical changes consequent to the use of NTAPP^50^.

Fibroblast and keratinocyte proliferation is important to restore tissue barrier function and for wound repair^51^. However, the IHOK and hTERT-hNOF proliferation rates on NTAPP-treated titanium were not significantly different than untreated samples. Therefore, it was evident that the chemical changes effected by NTAPP did not lead to enhancement of fibroblast^34^ and keratinocyte proliferation rates.

Finally, NTAPP treatment of titanium significantly reduced cellular inflammatory reactions compared to the control groups. Although inflammatory cytokine release is essential for body function, overstimulation of inflammatory cytokine release results in soft tissue destruction^18^. Therefore, as relevant in previous studies, cytokine levels should be reduced on biomaterial surfaces^52^. On the NTAPP-treated group compared with untreated titanium surfaces, cytokine release of inflamed soft tissue cells were reduced except for IL-8 from IHOKs. However, the inflamed cells on NTAPP-treated titanium exhibited high inflammatory cytokine release compared to that shown by normal cells. This suggests that NTAPP-treated titanium would not completely reduced cytokine release but rather may control the cytokine release necessary for proper inflammatory reactions. These findings indicate that NTAPP exposure on titanium could reduce the overstimulation of cytokine release of soft tissues, potentially decreasing tissue destruction. These results are due to reactive oxygen species (ROS) and reactive nitrogen species (RNS). ROS and RNS are already known as key role in the pathogenesis of periodontal disease^53–55^. Hence, ROS and RNS can be used as therapy of anti-inflammatory of soft tissue. Most cells can produce superoxide and hydrogen peroxide which belong to ROS that has significant role in cell signaling for determining cellular level^56,57^. However, there are two different conditions to be taken into account. ROS and RNS are used to improve the surface of Ti and they cannot directly stimulated oral gingival cells. ROS can improve Ti surface and improved Ti surface can stimulate cells.

According to results of this study, the first null hypothesis was fully rejected. This study indicated that the integration of normal soft tissue on titanium with and without NTAPP treatment showed significant differences. The second null hypothesis was partially rejected. There is a significant difference in cytokine release of inflamed soft tissue on NTAPP-treated titanium.

Despite the limitations of *in vitro* study, we found that NTAPP treatment with air gas on biomaterials such as titanium surface-based dental implant materials may potentially result in in favorable soft tissue integration and control of cytokine release. This positive effect may provide the basis for developing new strategies for NTAPP use in various biomaterials; this technique can ensure both excellent integration of soft tissue and control of cytokine release of inflamed gingival tissue surrounding dental implant abutment. Further study for *in vivo* or pre-clinical use is required to evaluate its effects.
